# Predictors of Severity in Covid-19 Patients in Casablanca, Morocco

**DOI:** 10.7759/cureus.10716

**Published:** 2020-09-29

**Authors:** Karim El Aidaoui, Amal Haoudar, Mohamed Khalis, Aziza Kantri, Jihane Ziati, Adil El Ghanmi, Ghita Bennis, Khalid El Yamani, Nezha Dini, Chafik El Kettani

**Affiliations:** 1 Anesthesia and Critical Care, Cheikh Khalifa International University Hospital, Mohammed VI University of Health Sciences, Casablanca, MAR; 2 Epidemiology and Public Health, Mohammed VI University of Health Sciences, Casablanca, MAR; 3 Mother and Child Department, Cheikh Khalifa International University Hospital, Mohammed VI University of Health Sciences, Casablanca, MAR

**Keywords:** predictive factors, severe covid-19, sars-cov-2, icu patients, hydroxychloroquine

## Abstract

Background

Morocco was affected, as were other countries, by the coronavirus disease 2019 (COVID-19) pandemic. Many risk factors of COVID-19 severity have been described, but data on infected patients in North Africa are limited. We aimed to explore the predictive factors of disease severity in COVID-19 patients in a tertiary hospital in Casablanca.

Methods

In this single-center, retrospective, observational study, we included all adult patients with confirmed severe acute respiratory syndrome coronavirus 2 (SARS-CoV-2) infection, admitted to Sheikh Khalifa International University Hospital in Casablanca between March 18 and May 20, 2020. Patients were separated into two groups: Non-severe patients were those with mild or moderate forms of COVID-19, and severe patients were those admitted to the intensive care unit (ICU) who had one of the following signs-respiratory rate > 30 breaths/min; oxygen saturation < 93% on room air; acute respiratory distress syndrome (ARDS); or required mechanical ventilation. Demographic, clinical, laboratory data, and outcomes were reviewed. We used univariable and multivariable logistic regression to explore predictive factors of severity.

Results

We reported 134 patients with confirmed SARS-CoV-2 infection. The median age was 53 years (interquartile range [IQR], 36-64), and 73 (54.5%) were men. Eighty-nine non-severe patients (66.4%) were admitted to single bedrooms, and 45 (33.6%) were placed in the ICU. The median time from illness onset to hospital admission was seven days (IQR, 3.0-7.2). Ninety-nine patients (74%) were admitted directly to the hospital, and 35 (26%) were transferred from other structures. Also, 68 patients (65.4%) were infected in clusters. Of the 134 patients, 61 (45.5%) had comorbidities, such as hypertension (n = 36; 26.9%), diabetes (n = 19; 14.2%), and coronary heart disease (n = 16; 11.9%). The most frequent symptoms were fever (n = 61; 45.5%), dry cough (n = 59; 44%), and dyspnea (n = 39; 29%). A total of 127 patients received hydroxychloroquine and azithromycin (95%). Eleven critical cases received lopinavir/ritonavir (8.2%). Five patients received tocilizumab (3.7%). We reported 13 ARDS cases in ICU patients (29%), eight with acute kidney injury (17.8%), and four thromboembolic events (8.8%). Fourteen ICU patients (31.1%) died at 28 days. In univariable analysis, older men with one or more comorbidities, infection in a cluster, chest scan with the COVID-19 Reporting and Data System (CO-RADS) 5, lymphopenia, high rates of ferritin, C-reactive protein (CRP), D-dimer, and lactate dehydrogenase were associated with severe forms of COVID-19. Multivariable logistic regression model founded increasing odds of severity associated with older age (odds ratio [OR] 1.05, 95% confidence interval [CI] 1.01-1.09, P = .0039), men (OR 3.19, CI 1.06-9.60, P = .016), one or more comorbidities (OR 4.36, CI 1.32-14.45, P = .016), CRP > 10 mg/L (OR 5.47, CI 1.57-19.10, P = .008), and lymphopenia lower than 0.8 x10^9^/L (OR 6.65, CI 1.43-30.92, P = .016).

Conclusions

Clinicians should consider older male patients with comorbidities, lymphopenia, and a high CRP rate as factors to predict severe forms of COVID-19 earlier. The higher severity of infected patients in clusters must be confirmed by epidemiological and genetic studies.

## Introduction

In March 2020, the first case of confirmed coronavirus disease 2019 (COVID-19) was registered in the Kingdom of Morocco; the patient who presented with acute pneumonia was an imported case from Europe. Since then and until August 5, a total of 27,217 laboratory-confirmed cases of infection with severe acute respiratory syndrome coronavirus 2 (SARS-CoV-2) have been reported in the different regions of the country, including 417 deaths. During the period from March to April 2020, while several northern Mediterranean countries (Spain, France, and especially Italy) were strongly affected by the epidemic [1], North Africa remained relatively spared with a limited number of patients admitted to intensive care units (ICU). As previously described in the literature, advanced age, comorbidities [2], and elevated inflammatory markers [3] are predictive factors of COVID-19 severity. Identifying these risk factors could help target vulnerable people in care strategies and reduce the mortality rate. But data on severely ill patients infected with SARS-CoV-2 in Africa are limited, especially in the north of the continent.

The Kingdom of Morocco is divided into 12 administrative regions, and the region of Casablanca is the largest and covers a population of 6,862,000 inhabitants [4]. In the Casablanca region during this epidemic, patients infected with SARS-CoV-2 are treated in dedicated health facilities through a secondary and tertiary health-care system that includes two tertiary care hospitals and eight secondary health-care clinics and hospitals. Patients with severe or critical clinical features of COVID-19 are treated in the two tertiary university hospitals: Sheikh Khalifa International University Hospital (SKIUH), which is a part of Mohammed VI University of Health Sciences (UM6SS), and Ibn Rochd Casablanca University Hospital. The Casablanca SKIUH has dedicated a set of units for COVID-19 patients: one isolation and diagnostic unit, two ICUs (23 beds), and a hospitalization unit with 42 single bedrooms. The objective of this study was to explore predictors of severity and describe the clinical course and outcomes of patients with COVID-19 disease, admitted to a tertiary hospital in Casablanca.

## Materials and methods

Study design

This single-center, retrospective, observational study was accepted by the Institutional scientific and ethics committees of SKIUH and UM6SS, and the use of patient data has been approved. This study describes the demographic characteristics, clinical presentation, coexisting conditions, imaging findings, and outcomes of incident cases of COVID-19 admitted to SKIUH from March 18, 2020, the date of the first confirmed case in our hospital, until May 20, 2020. This time frame was chosen to have a minimum follow-up of 15 days for all patients. The last patient in the series was admitted on April 20, 2020.

Participants and eligibility criteria

Only adult patients (≥18 years old) hospitalized for confirmed SARS-CoV-2 infection were included. A confirmed case of COVID-19 was defined by a positive result on a reverse transcriptase-polymerase chain reaction (RT-PCR) assay of a specimen collected on a nasopharyngeal swab. Only laboratory-confirmed cases were included. We separated the patients into two groups: severe patients and non-severe patients. Severe ill patients were defined as those admitted to the ICU who had one of the following signs: respiratory rate > 30 breaths/min; oxygen saturation < 93% on room air; acute respiratory distress syndrome (ARDS); or required mechanical ventilation. Non-severe patients were those with mild or moderate forms of COVID-19, according to the World Health Organization interim guidance [5].

Data collection

Electronic medical record data were collected through research on institutional software: DxCare SIH-HCK version 7.7-7p059 (Dedalus, France) and LIMS SGL-HCK version 11.4.3-26122018 (eNOVA). We obtained demographic data, coexisting conditions, clinical symptoms, and laboratory and radiologic results at the admission and during the hospital stay. All laboratory tests and radiologic assessments were performed at the discretion of the treating physicians. Any missing or uncertain records were collected and clarified through direct communication with involved health-care providers and family members of patients.

Statistical analysis

Descriptive statistics are provided by cross-tabulations with medians (interquartile ranges [IQR]) for continuous variables and frequencies (percentages) for qualitative variables. To compare the difference between the ICU and non-ICU groups, we used Student's t-test or Mann-Whitney U test for continuous variables and chi-squared test or Fisher's exact test for categorical variables, when appropriate. Univariable and multivariable logistic regression models were used to determine potential predictive factors of clinical severity in our patients. In the multivariable analysis, we included only significant variables with missing data of <30%. Variables with p values < .05 in univariable analysis were selected to be included in the multivariable logistic regression model. The final model included the following variables: age, sex, one or more comorbidities, C-reactive protein (CRP), and lymphocytes. Missing values for lymphocytes and CRP were treated as an additional variable category and were included in the final model. For all tests, a two-sided α < .05 was considered statistically significant (p value). Statistical analyses were done using IBM SPSS Statistics for Windows, version 26.0. (Armonk, NY: IBM Corp.).

## Results

Demographic and clinical characteristics

In this study, we reported 134 patients with confirmed SARS-CoV-2 infection. The median age was 53 years (IQR, 36-64), and 73 (54.5%) were men. Eighty-nine non-severe patients (66.4%) were admitted to single bedrooms, and 45 (33.6%) were placed in the ICU because of the development of organ dysfunction (Table 1). The time from illness onset to hospital admission was seven days (IQR, 3.0-7.2). 

**Table 1:** Demographic and clinical characteristics of patients Abbreviations: bpm, beats per minute; Diast. Art. Pres., diastolic arterial pressure; GCS, Glasgow Coma Scale; ICU, intensive care unit; SOFA, sequential organ failure assessment; Syst. Art. Pres., systolic arterial pressure. p values were calculated by Student’s t-test or Mann–Whitney U test for continuous variables and the chi-squared test or Fisher’s exact test for categorical variables. ^1^ Expressed in median (quartiles) ^2^ Expressed in frequency (%) ^3^ Expressed in mean ± standard deviation

Characteristics	Total (n = 134)	ICU (n = 45)	Non-ICU (n = 89)	p value
Age (years)^1^	53 (36-64)	64 (57-74)	42 (29-56)	< .001
Sex^2^				
Male	73 (54.5%)	35 (77.8%)	38 (42.7%)	< .001
Female	61 (45.5%)	10 (22.2%)	51 (57.3%)	
Time from illness onset to admission^1^	7 (3.0-7.2)	3 (2-7)	7 (4-8)	.155
Admission^2^				
Direct	99 (74%)	21 (46.7%)	64 (71.9%)	.004
Transfer	35 (26%)	24 (53.3%)	25 (28.1%)	
Cluster^2^				
Yes	75 (56%)	31 (68.9%)	44 (49.4%)	.032
No	59 (44%)	14 (31.1%)	45 (50.6%)	
Comorbidities^2^ ≥ one	61 (45.5%)	33 (73.3%)	28 (31.5%)	< .001
Diabetes	19 (14.2%)	12 (26.7%)	7 (7.9%)	.003
Hypertension	36 (26.9%)	22 (48.9%)	14 (15.7%)	< .001
Cardiac disease	16 (11.9%)	13 (28.9%)	3 (3.4%)	< .001
Asthma	10 (7.5%)	5 (11.1%)	5 (5.6%)	.303
Smoking	8 (5.2%)	4 (8.9%)	4 (4.5%)	.441
Chronic kidney disease	3 (2.2%)	3 (6.7%)	0	.036
Malignancy	2 (1.5%)	1 (2.2%)	1 (1.1%)	1.000
Chronic liver disease	1 (0.7%)	1 (2.2%)	0	.336
Ischemic stroke	1 (0.7%)	1 (2.2%)	0	.336
Signs and symptoms^2^				
Asthenia	25 (18.7%)	22 (49%)	3 (3.4%)	< .001
Fever	61 (45.5%)	27 (60%)	34 (38.2%)	.017
Headache	21 (15.7%)	5 (11%)	16 (18%)	.302
Myalgia	30 (22.4%)	7 (15.6%)	23 (25.8%)	.177
Rhinitis	8 (6%)	3 (6.7%)	5 (5.6%)	1.000
Pharyngalgia	10 (7.5%)	5 (11%)	5 (5.6%)	.303
Ageusia	28 (21%)	7 (15.6%)	23 (25.8%)	.280
Anosmia	25 (18.7%)	5 (11%)	20 (22.5%)	.111
Dry cough	59 (44%)	24 (53.3%)	35 (39.3%)	.123
Dyspnea	39 (29%)	25 (55.6%)	14 (15.7%)	< .001
Chest pain	5 (3.7%)	2 (4.4%)	3 (3.4%)	1.000
Abdominal pain	18 (13.4%)	5 (11%)	13 (14.6%)	.575
Diarrhea	29 (21.6%)	11 (24.4%)	18 (20.2%)	.575
Vomiting	22 (16.4%)	6 (13.3%)	16 (18%)	.493
Asymptomatic	20 (15%)	1 (2.2%)	19 (21.3%)	.003
Physical signs				
GCS (/15)^1^	15 (15-15)	15 (15-15)	15 (15-15)	.046
Respiratory rate (count/min)^3^	17 ± 5	22 ± 5	14 ± 1.5	< .001
Oxygen saturation (%)^3^	95 ± 5	90 ± 5	98 ± 1	< .001
Heart rate (bpm)^3^	82 ± 12	85 ± 16	82 ± 10	.162
Sys. Art. Pres. (mmHg)^3^	128 ± 14	133 ± 21	125 ± 5	.001
Diast. Art. Pres. (mmHg)^3^	67 ± 9	72 ± 12	65 ± 4	< .001
Quick SOFA^2^ ≥ 1	19 (14.2%)	19 (42.2%)	0	< .001

Ninety-nine patients (74%) were admitted directly to the hospital, and 35 (26%) were transferred from other structures. Also, 68 patients (65.4%) were infected in clusters. Of the 134 patients, 61 (45.5%) had comorbidities, such as hypertension (n = 36; 26.9%), diabetes (n = 19; 14.2%), and coronary heart disease (n = 16; 11.9%). The most frequent symptoms reported were fever (n = 61; 45.5%), dry cough (n = 59; 44%), dyspnea (n = 39; 29%). Other symptoms were myalgia, asthenia, headache, anosmia, ageusia, and digestive signs (Table 1).

In the group of the 45 ICU patients, the median age (64 years, IQR, 57-74) was higher significantly (p < .001) in comparison with the group not admitted to the ICU. Also, men were more frequent in the ICU group (n = 35; 77.8%) than in the non-ICU group (n = 38; 42.7%; p < .001). The median time from illness onset to hospital admission was three days (IQR, 2-7) for severe patients versus seven days (IQR, 3-7) for non-severe patients (the difference was not significant). There were more patients with comorbidities in the ICU group, including diabetes (n = 12; 26.7% versus n = 7; 7.9%), hypertension (n = 22; 48.9% versus n = 14; 15.7%), and cardiac disease (n = 13; 28.9% versus n = 3; 3.4%). This difference was significant (Table 1). Also, we have noticed that patients infected in a cluster were more severe significantly (n = 31; 68.9% versus n = 44; 49.4%). Patients in ICU presented higher incidence of fever (60% versus 38.2%), asthenia (49% versus 3.4%), dyspnea (55.6% versus 15.7%), and a lower incidence of asymptomatic forms (n = 1; 2.2% versus n = 19; 21.3%). These differences were significant. Regarding clinical examination parameters, there was a significant difference, especially in respiratory rate (22 ± 5 versus 14 ± 1.5) and oxygen saturation (90 ± 5 versus 98 ± 1) between ICU and non-ICU patients. The quick sequential organ failure assessment (SOFA) was ≥1 in 19 patients (14.2%); all of them were in the ICU group (42.2%).

Radiological and laboratory findings

In laboratory findings, there was a significant difference between severe patients and non-severe ones, including a higher rate of ferritin, D-dimers, CRP, lactate dehydrogenase (LDH), neutrophil counts, and a lower rate of lymphocytes in the ICU group (Table 2). 

**Table 2:** Radiological and laboratory findings on admission Abbreviations: ALAT, alanine aminotransferase; ASAT, aspartate aminotransferase; CO-RADS, COVID-19 Reporting and Data System; CRP, C-reactive protein; ICU, intensive care unit; LDH, lactate dehydrogenase. p values were calculated by Student’s t-test or Mann–Whitney U test for continuous variables and the chi-squared test or Fisher’s exact test for categorical variables. ^1^ Expressed in frequency (%) ^2^ Expressed in median (quartiles)

Radiological and laboratory findings	Total (n = 134)	ICU (n = 45)	Non-ICU (n = 89)	p value
Chest scan CO-RADS 4^1^	19 (14.2%)	8 (17.8%)	11 (12.4%)	.396
Chest scan CO-RADS 5^1^	31 (26.9%)	23 (51.1%)	8 (9%)	< .001
Ferritin^2^	174 (68-464)	645 (383-2289)	130 (43-233)	< .001
>300 µg/L (n = 96)	26/96 (26.3%)	16/21 (76.2%)	10/75 (13.3%)	< .001
D-dimer^2^	0.54 (0.30-0.87)	0.82 (0.54-2.44)	0.42 (0.26-0.68)	.001
>500 µg/L (n = 84)	44/84 (52.3%)	17/20 (85%)	27/64 (42.2%)	.001
LDH^2^	228 (178-281)	330 (254-399)	215 (164-252)	< .001
>250 U/L (n = 90)	32/90 (35.5%)	16/20 (80%)	16/70 (22.9%)	< .001
CRP^2^	11.8 (2.8-71.2)	86 (22-148)	3.7 (1.7-16.0)	< .001
>10 mg/L (n = 132)	69/132 (52.3%)	38/44 (86.4%)	31/88 (35.2%)	< .001
Lymphocytes^2^	1.45 (0.98-2.02)	0.99 (0.71-1.31)	1.65 (1.21-2.14)	< .001
<0.8 x 10^9^/L (n = 127)	21/127 (16.5%)	17/40 (42,5%)	4/87 (4.6%)	< .001
Neutrophils^2^ (x10^9^/L)	4.03 (2.68-5.45)	4.85 (3.45-6.84)	3.73 (2.37-4.89)	.001
White blood cell^2^ (x10^9^/L)	6.34 (4.72-7.40)	6.64 (5.41-8.22)	5.87 (4.5-7.26)	.06
Platelets^2^ (x10^9^/L)	266 (225-339)	263 (218-381)	270 (226-320)	.54
Hemoglobin^2^ (g/dL)	14.0 (12.8-15.0)	14.1 (12.6-14.9)	13.8 (12.8-15.0)	.77
Procalcitonin^2^ (ng/ml) (n = 113)	0.05 (0.05-0.31)	0.30 (0.10-0.45)	0.05 (0.05-0.05)	.002
ASAT^2^ (U/L) (n = 105)	28 (18-33)	34 (24-63)	20 (17-26)	< .001
ALAT^2^ (U/L) (n = 105)	22 (15-35)	35 (20-55)	19 (14-29)	< .001

On the imagery level, we adopted the COVID-19 Reporting and Data System (CO-RADS) to assess the degree of suspicion of the disease. Fully 74% of chest scans done on admission in severe patients were classified CO-RADS 5 compared to only 25.8% in non-severe patients.

Treatment and outcomes

Most of the 134 patients received antiviral therapy, including hydroxychloroquine (127 patients; 95%) and azithromycin (n = 127; 95%) with no difference between ICU and non-ICU patients. Critical cases of ICU patients had received lopinavir/ritonavir (n = 11; 24.4%). Five patients in the ICU group received tocilizumab (11.1%). Most of the patients received low molecular weight heparin (LMWH). LMWH was given at therapeutic dose for 43 patients of the ICU group (95%) and prophylaxis dose for 68 patients of the non-ICU group (76.4%). Corticosteroids were used in 14 severe cases (31%), and 22 patients in the ICU group (49%) received intravenous antibiotic therapy (Table 3). All patients also received vitamin C, zinc, and vitamin D supplementation as part of a national protocol.

**Table 3:** Treatment and outcomes Abbreviations: AKI, acute kidney injury; ARDS, acute respiratory distress syndrome; ICU, intensive care unit. p values were calculated by the chi-squared test or Fisher’s exact test for categorical variables. * Expressed in frequency (%).

Treatment and outcomes	Total (134)	ICU (n = 45)	Non-ICU (89)	p value
Complications*				
ARDS	13 (9.7%)	13 (28.9%)	0	< .001
AKI	8 (6%)	8 (17.8%)	0	< .001
Extrarenal epuration	8 (6%)	8 (17.8%)	0	< .001
Arrythmia	3 (2.2%)	3 (6.6%)	0	.029
Pneumothorax	2 (1.5%)	2 (4.4%)	0	.094
Thromboembolic events	4 (2.9%)	4 (8.8%)	0	.012
Septic shock	1 (0.01%)	1 (2.2%)	0	.303
Death	14 (10.4%)	14 (31.1%)	0	< .001
Causes of death*				
ARDS + AKI	8 (5.9%)	8 (17.8%)	0	< .001
ARDS alone	4 (3%)	4 (8.9%)	0	.012
Septic shock	1 (0.7%)	1 (2.2%)	0	.303
Decompensated cirrhosis	1 (0.7%)	1 (2.2%)	0	.198
Treatments*				
Hydroxychloroquine	127 (95%)	43 (95.5%)	84 (96.6%)	1.000
Azithromycin	127 (95%)	43 (95%)	84 (94.4%)	1.000
Lopinavir + ritonavir	11 (8.2%)	11 (24.4%)	0	.001
Tocilizumab	5 (3.7%)	5 (11.1%)	0	.004
Cephalosporin third generation	24 (17.9%)	22 (48.9%)	2 (2.2%)	< .001
Quinolones	21 (15.7%)	20 (44.4%)	1 (1.1%)	< .001
Aminosides	9 (6.7%)	9 (20%)	0	< .001
Carbapenem	8 (6%)	8 (17.8%)	0	< .001
Corticosteroids	14 (10.4%)	14 (31.1%)	0	< .001
Low molecular weight heparin	111 (82%)	43 (95.6%)	68 (76.4%)	.005
Catecholamines	13 (9.7%)	13 (28.9%)	0	< .001
Non-rebreather mask	23 (17.2%)	23 (51.1%)	0	< .001
Noninvasive ventilation	8 (5.9%)	8 (17.8%)	0	< .001
Invasive mechanical ventilation	14 (10.4%)	14 (31.1%)	0	< .001

Complications were associated with severe and critical patients. We reported the following cases in the 45 ICU patients: 13 ARDS (28.9%), eight acute kidney injury (AKI; 17.8%), four confirmed thromboembolic events (8.8%), three arrhythmias (6.6%), and two pneumothorax (4.4%). Fourteen patients of the ICU group (31.1%) required invasive mechanical ventilation, of whom 11 patients (78%) died. Also, 14 patients among those admitted to ICU died at 28 days (31.1%). Causes of death for ICU patients were mainly due to an association of ARDS and AKI (n = 8; 17.8%) or ARDS alone (n = 4; 8.9%). One patient died of septic shock, and one patient died of decompensated cirrhosis (Table 3).

Univariable and multivariable analysis

In univariable analysis, the odds ratio (OR) of severity was higher in older men with hypertension, diabetes, cardiac disease, infection in a cluster, and chest scan CO-RADS 5. High rates of biological markers such as ferritin, CRP, D-dimer, and LDH were associated with severe forms of COVID-19, as well as a rate of lymphocytes < 0.8 x 10^9^/L (Table 4). In the multivariable logistic regression model, we included five variables, which were significant with missing data < 30%. We founded increasing OR of severity associated with older age (OR = 1.05, 95% CI 1.01-1.09) per year increase, p = .0039), men (OR = 3.19, CI 1.06-9.60, p = .016), one or more comorbidities (OR = 4.36, CI 1.32-14.45, p = .016), CRP > 10 mg/L (OR = 5.47, CI 1.57-19.10, p = .008), and lymphopenia < 0.8 x 10^9^/L (OR = 6.65, CI 1.43-30.92, p = .016).

**Table 4:** Predictive factors associated with severe forms of COVID-19 (logistic regression) Abbreviations: CI, confidence interval; CO-RADS, COVID-19 Reporting and Data System; CRP, C-reactive protein; LDH, lactate dehydrogenase; OR, odds ratio.

Predictive factors	Univariable analysis			Multivariable analysis		
Characteristics	OR	CI (95%)	p value	OR	CI (95%)	p value
Age	1.09	1.05-1.12	< .001	1.05	1.01-1.09	.039
Sex						
Female	1 (ref)					
Male	4.70	2.07-10.65	< .001	3.19	1.06-9.60	.016
Comorbidities	5.99	2.70-13.30	< .001	4.36	1.32-14.45	.016
Hypertension	5.12	2.26-11.60	< .001			
Diabetes	4.26	1.54-11.76	.005			
Cardiac disease	11.64	3.11-43.56	< .001			
Cluster						
No	1 (ref)					
Yes	2.26	1.06-4.82	.034			
Chest scan CO-RADS 5						
No	1 (ref)					
Yes	10.58	4.16-26.89	< .001			
Ferritin (µg/L)	1.003	1.002-1.005	< .001			
≤300	1 (ref)					
>300	19.50	5.80-65.49	< .001			
D-dimer (µg/L)	2.47	1.33-4.58	.004			
≤500	1 (ref)					
>500	7.76	2.07-29.18	.002			
LDH (U/L)	1.015	1.007-1.023	< .001			
≤250	1 (ref)					
>250	13.50	3.95-46.16	< .001			
CRP (mg/L)	1.023	1.013-1.033	< .001			
≤10	1 (ref)					
>10	11.64	4.43-30.59	< .001	5.47	1.57-19.10	.008
Lymphocytes (10^9^/L)	0.126	0.052-0.307	< .002			
≥0.8	1 (ref)					
<0.8	15.34	4.70-50.06	< .001	6.65	1.43-30.92	.016

## Discussion

We report on 134 patients with confirmed SARS-CoV-2 infection, of whom 45 (33%) were characterized by severe hypoxemia and admitted to the ICU. This study adds supplementary data about specifics of COVID 19 in Morocco and North Africa. Overall, data from a few thousand analyzed patients show that severe illness can be expected in the elderly [6,7], although the younger patients are not protected either. The age range in severe patients in most Chinese studies is 52 to 66 years [2,8]. In the Italian population, the case-fatality rate increases with age: 12% in patients older than 70 years and 20% in those older than 80 years [1]. In this study, we found that severe patients are older than non-severe ones, which is similar to published data from China and Italy. We also found that most severe patients are men, as demonstrated by Zheng et al. in a meta-analysis [9]. In our study, severe patients had more diabetes mellitus, hypertension, and cardiac diseases, as published by Huang et al. [3]. Fever, cough, and dyspnea were the most common symptoms in patients with severe COVID-19. A higher body temperature (>38°C) observed initially, and a high breathing rate also appeared to be predictive of worsening disease severity [9]. 

In this study, the average duration between the onset of symptoms and hospital admission is about seven days, and it was shorter in severe cases (3 days). Zhou et al. reported in their study that the median time from illness onset to dyspnea ranged from 4 to 9 days [10]. So, clinicians should be aware of the potential for some patients to rapidly deteriorate one week after the onset of symptoms. We found also a significantly higher frequency of infected patients in the context of a family or religious cluster in the ICU patient group. We have no explanation for this finding, and our hypotheses are either contamination by a large inoculum in the context of a cluster or a genetic predisposition within certain families to present severe forms of this viral disease. Epidemiological, immunological, and genetic studies are needed to explain and to confirm that. Regarding the radiological investigations, computed tomography (CT) imaging has a high sensitivity for the diagnosis of severe COVID-19 pneumonia [11]. For practical reasons, we adopted the CO-RADS classification developed by the Dutch Radiological Society [12]. This classification assesses the suspicion for pulmonary involvement of COVID-19 on a scale from 1 to 5. We found that images classified CO-RADS 5 in the chest scanner were significantly correlated with the severity of the COVID-19.

Regarding the laboratory data in our series, specific biological parameters were predictive of the severity of the clinical outcome of our patients, such as elevation of LDH, ferritin, D-dimers, CRP, neutrophils, and lymphopenia. This is consistent with the data reported by Rodriguez et al., who raised in a meta-analysis the presence of frequent biological abnormalities such as elevated levels of inflammatory markers (CRP, LDH) and lymphopenia [13]. The frequency of lymphopenia observed by us and others suggests that COVID-19 could act on lymphocytes, particularly T lymphocytes, with perhaps a depletion of CD4 and CD8 cells. This notion has been demonstrated in severe acute respiratory syndrome (SARS) [14]. Henry et al., in a meta-analysis, used the number of lymphocytes, particularly CD4 cells, as a biological predictor of severity and prognosis. They reported the hypothesis that survival might depend on the ability to restore lymphocytes that are killed by the virus in the case of COVID-19 [15]. Henry et al. also reported very significant increases in ferritin and CRP in severe patients [15], which is consistent with data collected from our patients. The increase in CRP reflects the magnitude of the systemic inflammatory syndrome present in severe forms of the disease. The latter is believed to result in a massive release of the inflammatory cytokines creating a "cytokine storm" responsible for acute tissue damage with the onset of ARDS, in addition to subsequent multi-systemic failure [15]. Elsewhere, the increase in LDH levels observed in our series is consistent with the outcome observed by the other teams who have correlated LDH, lymphocytes, neutrophils, and CRP abnormalities with the acute pulmonary involvement characteristic of severe forms [16]. Markers of inflammation, such as CRP, ferritin, and interleukin-6, are also significantly associated with mortality [2].

In this study, patients with a severe form of COVID-19 pneumonia had significantly higher neutrophil counts than non-severe patients. Guan et al. reported that COVID-19-related multiple-organ failure and ARDS are due to cytokine storm caused by neutrophils [11]. The severity of lung damage was correlated with extensive pulmonary infiltration of neutrophils and macrophages and high levels of these cells in the peripheral blood in patients with Middle East respiratory syndrome (MERS) [17,18]. The generation of cytokine storm due to neutrophils can lead to ARDS, which is a leading cause of death in patients with COVID-19 [17], as it was previously seen in MERS [18] and SARS [19] . Post-viral hyperinflammation with onset on the second week of the disease mostly seems to explain disease severity [20]. The question, however, is how can we identify in time the patients who are at risk of rapid deterioration.

Of all included patients, 45 patients (33%) had severe or critical condition, which was similar to the Italian data that showed 30% of COVID-19 patients were severe and 5% were critically ill [1]. In data from China, 5% of all COVID-19 patients were placed in an ICU [11]. Fourteen patients died at 28 days (31% of severe ones and 10.4% of all patients); this is higher than mortality rates found in Chinese and Italian data [1,11]. This is likely due to the high transfer of severe patients to our hospital (53% of transferred patients were admitted to ICU). Several Chinese studies report very high mortality rates for intensive care (81% to 97%) and those requiring mechanical ventilation among patients [7,8]. In the ICU group, one-third of patients had critical ARDS and required mechanical ventilation. This rate is similar to that found in the meta-analysis of Cao et al. [21]. Eleven of those patients with ARDS died (78%). It was the primary cause of death in this study.

The second complication in the ICU patients was AKI requiring hemodialysis with 17.8%. This rate is higher than that found in Cheng et al. [22]. This study demonstrated that the development of AKI during hospitalization in patients with COVID-19 is associated with in-hospital mortality. We also had 8.8% of confirmed thromboembolic events, but this rate is probably low and does not reflect the frequency of venous thromboembolic events in this group of patients. Indeed, for health-care workers’ safety, we have not performed screening Doppler ultrasound of the lower limbs or systematic chest CT for any worsening of patients' respiratory status. Estimates of the risk of arterial and venous thromboembolic complications are still preliminary and depend on the local diagnosis and pharmacological preventive strategies [23]. However, It has been postulated that the high mortality observed among COVID-19 patients may be partly due to unrecognized pulmonary embolism and pulmonary in situ thrombosis [23]. That is why all patients of the ICU group received LMWH at therapeutic dose. We also had two pneumothorax in two ventilated patients, but barotraumas seem less severe in patients with SARS-CoV-2 infection who are being mechanically ventilated than in patients with SARS-CoV-1 infection who are being mechanically ventilated [8]. Barotraumas occurred in about 25% of patients with SARS-CoV-1 on mechanical ventilation [8]. The lower occurrence of barotraumas in this study is probably related to the strategy of protective ventilation.

Because the pathogenesis of highly pathogenic human coronavirus is still not completely understood, and without substantial evidence, most patients were given antiviral agents: association of chloroquine and azithromycin in the first intention or lopinavir/ritonavir in the second intention (for severe forms in case of non-clinical improvement in the fifth day). In case of bacterial infection, we gave a first line antibiotic therapy (cephalosporin third generation and quinolones), and in case of no effectiveness after 48 h, we switched on carbapenem. All patients with severe COVID-19 should be screened for hyperinflammation using laboratory trends to identify patients for whom immunosuppression could improve mortality [24]. In this context, tocilizumab seems to be an effective option in COVID-19 patients with a risk of cytokine storm in order to reduce the severity and/or mortality of the disease [25]. Five severe cases in this study with increasing inflammatory markers received tocilizumab but without the biological assay of interleukin-6, which was not available. Three of the five patients survived. Because of incomplete evidence, corticosteroids are not routinely recommended during epidemics of SARS and MERS and might exacerbate COVID-19-associated lung injury [26]. In this study, one-third of severe patients who developed ARDS on disease progression were given intravenous corticosteroids (methylprednisolone at a daily dose of 1 mg/kg) without convincing results.

This study has notable limitations. First, we may have included disproportionately more patients with poor outcomes (due to the transfer of patients in serious condition to our hospital). Second, due to the retrospective study design, some cases had incomplete documentation of laboratory testing. Third, interpretation of our results might be limited by the sample size, but we think that our study population is representative of patients treated in Morocco and North Africa.

## Conclusions

In this single-center retrospective observational study of 134 patients with COVID-19, the average duration between the onset of symptoms and hospital admission was shorter in severe patients. Epidemiological and genetic studies must confirm the high frequency of infected patients in the context of a family or religious cluster among severe cases. Clinicians should consider older men patients with comorbidities, lymphopenia, and a high CRP rate to predict severe COVID-19 at an early stage of hospitalization.
